# Predictive model for sarcopenia in chronic kidney disease: a nomogram and machine learning approach using CHARLS data

**DOI:** 10.3389/fmed.2025.1546988

**Published:** 2025-03-12

**Authors:** Renjie Lu, Shiyun Wang, Pinghua Chen, Fangfang Li, Pan Li, Qian Chen, Xuefei Li, Fangyu Li, Suxia Guo, Jinlin Zhang, Dan Liu, Zhijun Hu

**Affiliations:** ^1^Longhua Clinical Medical College of Shanghai University of Traditional Chinese Medicine, Shanghai, China; ^2^Longhua Hospital Affiliated to Shanghai University of Traditional Chinese Medicine, Shanghai, China; ^3^Ruijin Hospital North Campus, Shanghai Jiao Tong University, Shanghai, China

**Keywords:** sarcopenia, chronic kidney disease, predictive model, nomogram, machine learning, CHARLS

## Abstract

**Background:**

Sarcopenia frequently occurs as a complication among individuals with chronic kidney disease (CKD), contributing to poorer clinical outcomes. This research aimed to create and assess a predictive model for the risk of sarcopenia in CKD patients, utilizing data obtained from the China Health and Retirement Longitudinal Study (CHARLS).

**Methods:**

Sarcopenia was diagnosed based on the Asian Working Group for Sarcopenia (AWGS 2019) criteria, including low muscle strength, reduced physical performance, and low muscle mass. The 2015 CHARLS data were split randomly into a training set (70%) and a testing set (30%). Forty-nine variables encompassing socio-demographic, behavioral, health status, and biochemical factors were analyzed. LASSO regression identified the most relevant predictors, and a logistic regression model was used to explore factors associated with sarcopenia. A nomogram was developed for risk prediction. Model accuracy was evaluated using calibration curves, while predictive performance was assessed through receiver operating characteristic (ROC) and decision curve analysis (DCA). Four machine learning algorithms were utilized, with the optimal model undergoing hyperparameter optimization to evaluate the significance of predictive factors.

**Results:**

A total of 1,092 CKD patients were included, with 231 (21.2%) diagnosed with sarcopenia. Multivariate logistic regression revealed that age, waist circumference, LDL-C, HDL-C, triglycerides, and diastolic blood pressure are significant predictors. These factors were used to construct the nomogram. The predictive model achieved an AUC of 0.886 (95% CI: 0.858–0.912) in the training set and 0.859 (95% CI: 0.811–0.908) in the validation set. Calibration curves showed good agreement between predicted and actual outcomes. ROC and DCA analyses confirmed the model’s strong predictive performance. The Gradient Boosting Machine (GBM) outperformed other machine learning models. Applying Bayesian optimization to the GBM achieved an AUC of 0.933 (95% CI: 0.913–0.953) on the training set and 0.932 (95% CI: 0.905–0.960) on the validation set. SHAP values identified age and waist circumference as the most influential factors.

**Conclusion:**

The nomogram provides a reliable tool for predicting sarcopenia in CKD patients. The GBM model exhibits strong predictive accuracy, positioning it as a valuable tool for clinical risk assessment and management of sarcopenia in this population.

## 1 Introduction

Sarcopenia, characterized by the progressive loss of skeletal muscle mass, strength, and functionality, poses an increasing health challenge, particularly for older adults and individuals with chronic illnesses. As kidney function declines, there is a notable deterioration in muscle health, which significantly affects physical capabilities and overall quality of life (1). Chronic kidney disease (CKD) affects millions worldwide and leads to various complications, including metabolic imbalances, nutritional deficiencies, and reduced physical capacity, all of which exacerbate muscle function decline (2–4). Sarcopenia frequently occurs as a complication among individuals with CKD, with a global prevalence of 24.5% across all disease stages (5). This progressive muscle deterioration contributes to poorer clinical outcomes, including increased mortality risk (6). While recent studies have developed nomograms for sarcopenia prediction in hemodialysis populations, reliable tools for non-dialysis CKD patients remain scarce. This gap underscores the need for models adaptable to broader CKD stages (7, 8). Developing an accurate risk prediction model would aid healthcare providers in identifying high-risk individuals early, facilitating the timely implementation of interventions focused on nutrition and physical rehabilitation. These interventions are crucial for enhancing the quality of life, minimizing the risk of additional health complications, and reducing overall healthcare costs.

The China Health and Retirement Longitudinal Study (CHARLS) provides a valuable opportunity to develop such a predictive model (9). It is a nationally representative survey of Chinese adults aged 45 and older, designed to collect comprehensive data on health, social, and economic factors (10). The dataset includes comprehensive information, such as biomarkers, physical measurements, and health histories, making it an ideal resource for developing models that predict adverse health outcomes associated with aging (11). The primary objective of this study is to develop and validate a predictive model for muscle health decline in patients with CKD using CHARLS data. The model is designed to enhance the early detection of high-risk individuals, facilitating prompt interventions to avert severe health decline.

## 2 Materials and methods

### 2.1 Study design and population

This study draws on data from CHARLS. The CHARLS dataset includes extensive details about participants’ demographic profiles, health conditions, socioeconomic status, and lifestyle habits. This study analyzes data obtained from the publicly accessible 2015 wave of CHARLS, which provides comprehensive health-related measurements.

**Figure 1:**
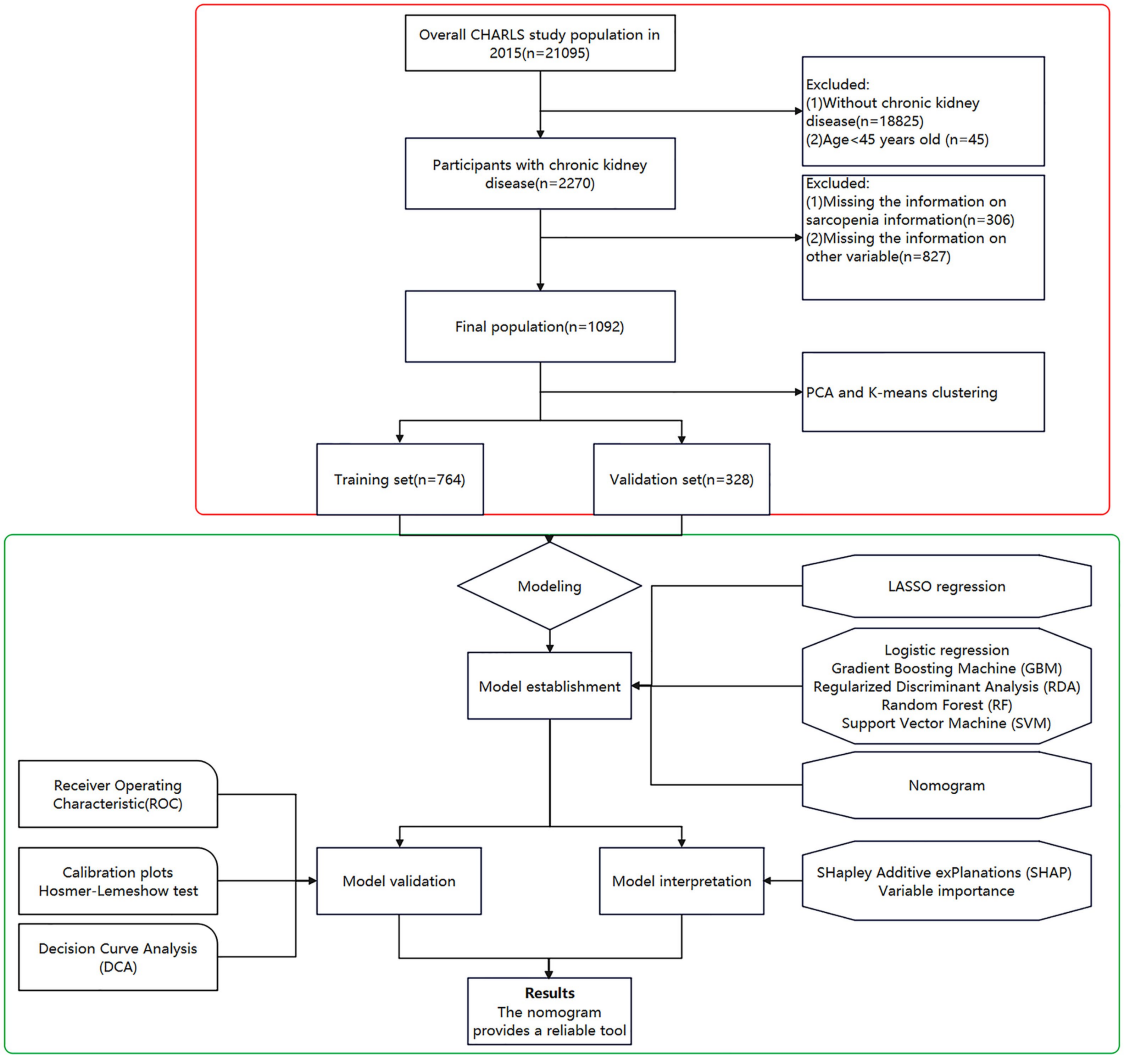
Flowchart of study design.

Participants eligible for inclusion in the study were individuals diagnosed with chronic kidney disease (CKD), identified through either self-reported confirmation by a physician or clinical diagnostic criteria, such as an estimated glomerular filtration rate (eGFR) of less than 60 mL/min/1.73 m^2^ (12). To uphold the robustness of the analysis, those with incomplete data on key variables—including sarcopenia-related metrics, renal function indicators, and other covariates integral to the predictive model—were excluded. Consequently, the final analytic sample comprised 1,092 CKD patients aged 45 years or older, each with comprehensive data suitable for evaluation (Figure 1).

### 2.2 Definition of sarcopenia

Sarcopenia status was evaluated in accordance with the 2019 criteria established by the Asian Working Group for Sarcopenia (AWGS), encompassing three core components: muscle strength, appendicular skeletal muscle mass (ASM), and physical performance. The diagnosis of sarcopenia was assigned to individuals exhibiting reduced muscle mass in conjunction with either compromised muscle strength or diminished physical performance (13). Muscle Strength was measured using a Yuejian™ WL-1000 dynamometer. Participants stood upright, holding the dynamometer at a right angle to their body, and were instructed to squeeze the handle as firmly as possible for 3 s. Two trials were performed for each hand, with a minimum rest interval of 15 s between consecutive trials to minimize fatigue. The highest value across all four trials (left and right hands combined) was recorded as the final grip strength. Consistent with the AWGS 2019 criteria, low grip strength was defined as <28 kg for men and < 18 kg for women.

ASM was estimated using a validated anthropometric equation specifically designed for Chinese residents. Previous studies have demonstrated that this equation yields results closely aligned with those obtained from dual X-ray absorptiometry (DXA) (14, 15). Low muscle mass was defined as the sex-specific lowest 20% of height-adjusted ASM (ASM/Ht^2^) within the cohort, consistent with methodologies applied in large-scale Chinese sarcopenia studies (16). Specifically, for women, low muscle mass was defined as less than 5.37 kg/m^2^, and for men, it was less than 7.06 kg/m^2^. The ASM equation utilized is:


$$\begin{array}{l} ASM=0.193*\mathrm{weight}\;\left(\mathrm{kg}\right)+0.107*\mathrm{height}\;\left(\mathrm{cm}\right)-4.157\\ {}*\mathrm{gender}\left(1=\mathrm{men},2=\mathrm{women}\right)-0.037*\mathrm{age}\;\left(\mathrm{years}\right)-2.631 \end{array}$$

Additionally, physical performance was assessed through gait speed and a 5-time chair stand test. For gait speed, participants completed two trials of a 2.5-meter walk at their usual pace, with timing initiated when the first foot crossed the start line and stopped at the finish line; the maximum speed from either trial was used, with <1 m/s defined as low performance. 5-Time Chair Stand Test required participants to rise five times as quickly as possible with arms crossed over the chest. Timing began on the command “ready? stand” and ended when the participant’s buttocks touched the chair after the fifth stand. A practice trial was allowed prior to formal measurement. Low performance was defined as a completion time ≥ 12 s (13).

### 2.3 Variables

#### 2.3.1 Demographic characteristics

The demographic factors considered in this study were age, gender, marital status, and education level. Marital status was categorized as either married or not married. Educational attainment was categorized into primary, secondary, or tertiary education levels.

#### 2.3.2 Lifestyle and behavioral factors

Lifestyle and behavioral factors encompassed smoking status, alcohol use, physical and social activities, sleep quality, and life satisfaction. Smoking status was categorized into two groups: current smoker and non-smoker. Alcohol consumption was categorized into two groups based on drinking history: individuals with a history of alcohol use and those without. Physical activity was categorized by frequency and intensity as high, moderate, or low. Social activity was classified into two categories based on engagement: those who participate in social activities and those who do not. Sleep quality was classified into four levels based on self-reported frequency of poor sleep: “Rarely or none of the time,” indicating poor sleep is infrequent or almost never occurs; “Some or a little of the time,” suggesting poor sleep occurs occasionally but not regularly; “Occasionally or a moderate amount of the time,” denoting that poor sleep is experienced somewhat regularly; and “Most or all of the time,” reflecting frequent or consistent poor sleep. Life satisfaction was measured and divided into three levels: “Fair,” indicating average life satisfaction; “Good,” indicating above-average satisfaction where individuals feel mostly content with their lives; and “Poor,” signifying below-average satisfaction, where individuals often feel dissatisfied.

#### 2.3.3 Health-related factors

Health-related factors included body index, self-perceived health status, ADL (Activities of Daily Living) disability, IADL (Instrumental Activities of Daily Living) disability, and depressive symptoms. Body index encompassed body mass index (BMI), waist circumference, height, and weight. BMI was determined by dividing weight in kilograms by height in meters squared. Self-reported health status was categorized as good, fair, or poor based on participants’ own assessment of their overall health. The ADL scale assesses participants’ ability to perform basic tasks such as dressing, bathing, feeding, transferring, toileting, and maintaining continence. If a participant is completely unable to perform any one of these tasks, they are considered to have an ADL disability. Similarly, the IADL scale evaluates more complex tasks, including doing chores, cooking, shopping, managing finances, and taking medication. If a participant is completely unable to perform any one of these tasks, they are considered to have an IADL disability (17–19). Depressive symptoms were assessed using a 10-item screening questionnaire derived from the Center for Epidemiologic Studies Depression Scale (CES-D), which evaluates depressive mood and behavior. A CES-D score greater than 10 was used to define the presence of depression (20, 21).

#### 2.3.4 Chronic diseases

Chronic diseases were identified through self-reported diagnoses, including mental disease, stroke, heart disease, cardiovascular disease, arthritis, dyslipidemia, liver disease, digestive disease, hypertension, chronic lung disease, and asthma.

#### 2.3.5 Laboratory test indicators

Laboratory test indicators included blood pressure, lipid profile, renal function markers, hematological indicators, and inflammatory markers. Blood pressure measurements included systolic and diastolic values. The lipid profile included low-density lipoprotein (LDL-C), triglycerides (TG), high-density lipoprotein (HDL-C), and total cholesterol (TC). Renal function markers included cystatin C, serum creatinine, blood urea nitrogen (BUN), and uric acid levels. The estimated glomerular filtration rate (eGFR) serves as an important indicator of kidney function. In this study, we used a formula based on serum cystatin C (Cys) levels, known for its accuracy. This method, derived from the CKD-EPI equation, provides a reliable assessment of kidney function, especially in populations where creatinine-based estimates are less accurate (22). Hematological indicators included glycated hemoglobin and C-reactive protein.

### 2.4 Data processing

The CHARLS 2015 dataset was randomly divided into two subsets: a training set (70% of the data) and an internal validation set (30% of the data). LASSO (Least Absolute Shrinkage and Selection Operator) regression was performed on the training set to identify possible predictors of sarcopenia in individuals with chronic kidney disease (CKD). Variables that showed significant associations were included in a subsequent multivariate logistic regression analysis, where independent predictors were determined using a significance level of *p* < 0.05. These identified predictors were then utilized to create a nomogram for evaluating sarcopenia risk (23).

### 2.5 Model performance assessment

The model’s ability to discriminate was assessed using the area under the receiver operating characteristic curve (AUC). Calibration was examined through the use of calibration plots and the Hosmer-Lemeshow test, which assessed how well the predicted probabilities aligned with the actual outcomes. To determine the clinical utility of the nomogram, Decision Curve Analysis (DCA) was performed.

### 2.6 Model development and evaluation

To evaluate the predictive performance of the identified predictors, four machine learning models were developed: Gradient Boosting Machine (GBM), Regularized Discriminant Analysis (RDA), Random Forest (RF), and Support Vector Machine (SVM). For further optimization, Bayesian optimization was employed for hyperparameter tuning. Additionally, SHapley Additive exPlanations (SHAP) values and variable importance metrics were used to assess feature contributions.

### 2.7 Principal component analysis and cluster analysis

Principal Component Analysis (PCA) was conducted to reduce dimensionality and mitigate multicollinearity (24). The analysis included all predictor variables, excluding ID and the outcome variable (sarcopenia). The number of principal components (PCs) retained was determined based on cumulative explained variance, with the goal of capturing at least 75% of the total variance. K-means clustering was performed to identify subgroups with similar characteristics. The optimal number of clusters was determined using the Elbow method and Silhouette coefficient. The clusters were analyzed in relation to sarcopenia prevalence to explore potential group-specific differences (25).

### 2.8 Statistical analysis

All statistical analyses were performed using R version 4.3.1. Continuous variables are expressed as mean ± standard deviation (SD) or median (interquartile range, IQR), depending on suitability, whereas categorical variables are shown as frequencies and percentages. Appropriate statistical tests, including Student’s t-test, chi-square test, and Mann–Whitney U test, were employed for group comparisons. A two-tailed *p*-value below 0.05 was deemed statistically significant.

## 3 Results

### 3.1 Study population characteristics

A total of 1,092 CKD patients were included in the final analysis, with 231 diagnosed with sarcopenia and 861 without, yielding a sarcopenia incidence of 21.2%. The demographic and clinical features of the sample, which were used for model development, are presented in Table 1. Notable differences were found between the groups in terms of age, marital status, BMI, and waist circumference (*p* < 0.05). Individuals with sarcopenia were generally older and exhibited a higher rate of comorbidities, including hypertension and dyslipidemia (*p* < 0.05).

**Table 1:** Baseline characteristics of samples.

Variables	Overall (*n* = 1,092)	Non-Sarcopenia (*n* = 861)	Sarcopenia (*n* = 231)	*p*-value
Age	65.41 (9.81)	63.99 (9.22)	70.74 (10.15)	<0.001
Gender (%)				0.01
Female	552 (50.5)	453 (52.6)	99 (42.9)	
Male	540 (49.5)	408 (47.4)	132 (57.1)	
Marital (%)				<0.001
Married	888 (81.3)	720 (83.6)	168 (72.7)	
Unmarried	204 (18.7)	141 (16.4)	63 (27.3)	
Education (%)				0.427
Primary	1,038 (95.1)	815 (94.7)	223 (96.5)	
Secondary	48 (4.4)	40 (4.6)	8 (3.5)	
Tertiary education	6 (0.5)	6 (0.7)	0 (0.0)	
Area (%)				0.01
Rural	921 (84.3)	713 (82.8)	208 (90.0)	
Urban	171 (15.7)	148 (17.2)	23 (10.0)	
Alcohol (%)				0.761
No	764 (70.0)	600 (69.7)	164 (71.0)	
Yes	328 (30.0)	261 (30.3)	67 (29.0)	
Smoking (%)				0.005
No	560 (51.3)	461 (53.5)	99 (42.9)	
Yes	532 (48.7)	400 (46.5)	132 (57.1)	
Socialactivity (%)				0.003
No	503 (46.1)	376 (43.7)	127 (55.0)	
Yes	589 (53.9)	485 (56.3)	104 (45.0)	
Sleep quality (%)				0.091
Rarely or none of the time	424 (38.8)	328 (38.1)	96 (41.6)	
Some or a little of the time	172 (15.8)	148 (17.2)	24 (10.4)	
Occasionally or a moderate amount of the time	181 (16.6)	139 (16.1)	42 (18.2)	
Most or all of the time	315 (28.8)	246 (28.6)	69 (29.9)	
Life satisfaction (%)				0.396
Fair	662 (60.6)	513 (59.6)	149 (64.5)	
Good	384 (35.2)	311 (36.1)	73 (31.6)	
Poor	46 (4.2)	37 (4.3)	9 (3.9)	
Health (%)				0.895
Fair	477 (43.7)	378 (43.9)	99 (42.9)	
Good	87 (8.0)	67 (7.8)	20 (8.7)	
Poor	528 (48.4)	416 (48.3)	112 (48.5)	
ADL_disability (%)				0.477
No	1,043 (95.5)	820 (95.2)	223 (96.5)	
Yes	49 (4.5)	41 (4.8)	8 (3.5)	
IADL_disability (%)				0.377
No	912 (83.5)	724 (84.1)	188 (81.4)	
Yes	180 (16.5)	137 (15.9)	43 (18.6)	
BMI	24.13 (4.52)	25.47 (4.07)	19.17 (1.86)	<0.001
Waist	85.98 (14.78)	89.19 (13.63)	74.03 (12.63)	<0.001
Height	157.30 (8.09)	157.72 (7.99)	155.70 (8.29)	0.001
Weight	59.83 (12.53)	63.40 (11.37)	46.51 (5.96)	<0.001
CESD	10.93 (7.27)	10.94 (7.23)	10.88 (7.44)	0.918
Depression (%)				1
No	541 (49.5)	427 (49.6)	114 (49.4)	
Yes	551 (50.5)	434 (50.4)	117 (50.6)	
Hypertension (%)				<0.001
No	597 (54.7)	435 (50.5)	162 (70.1)	
Yes	495 (45.3)	426 (49.5)	69 (29.9)	
Chronic lung diseases (%)				0.167
No	843 (77.2)	673 (78.2)	170 (73.6)	
Yes	249 (22.8)	188 (21.8)	61 (26.4)	
Cardiovascular disease (%)				0.037
No	739 (67.7)	569 (66.1)	170 (73.6)	
Yes	353 (32.3)	292 (33.9)	61 (26.4)	
Stroke (%)				0.343
No	1,028 (94.1)	807 (93.7)	221 (95.7)	
Yes	64 (5.9)	54 (6.3)	10 (4.3)	
Mental disease (%)				0.212
No	1,067 (97.7)	844 (98.0)	223 (96.5)	
Yes	25 (2.3)	17 (2.0)	8 (3.5)	
Arthritis (%)				0.093
No	460 (42.1)	351 (40.8)	109 (47.2)	
Yes	632 (57.9)	510 (59.2)	122 (52.8)	
Dyslipidemia (%)				<0.001
No	832 (76.2)	623 (72.4)	209 (90.5)	
Yes	260 (23.8)	238 (27.6)	22 (9.5)	
Liver disease (%)				0.437
No	965 (88.4)	757 (87.9)	208 (90.0)	
Yes	127 (11.6)	104 (12.1)	23 (10.0)	
Digestive disease (%)				0.986
No	660 (60.4)	521 (60.5)	139 (60.2)	
Yes	432 (39.6)	340 (39.5)	92 (39.8)	
Diabetes (%)				0.185
No	927 (84.9)	724 (84.1)	203 (87.9)	
Yes	165 (15.1)	137 (15.9)	28 (12.1)	
Asthma (%)				0.459
No	981 (89.8)	777 (90.2)	204 (88.3)	
Yes	111 (10.2)	84 (9.8)	27 (11.7)	
ADL_score	5.85 (0.53)	5.83 (0.57)	5.91 (0.30)	0.038
Cognition	9.70 (4.22)	10.08 (4.06)	8.26 (4.52)	<0.001
Hearing (%)				0.3
Fair	624 (57.1)	497 (57.7)	127 (55.0)	
Good	243 (22.3)	195 (22.6)	48 (20.8)	
Poor	225 (20.6)	169 (19.6)	56 (24.2)	
Vision (%)				0.647
Fair	491 (45.0)	393 (45.6)	98 (42.4)	
Good	318 (29.1)	246 (28.6)	72 (31.2)	
Poor	283 (25.9)	222 (25.8)	61 (26.4)	
Pain (%)				0.939
No	58 (5.3)	45 (5.2)	13 (5.6)	
Yes	1,034 (94.7)	816 (94.8)	218 (94.4)	
eGFR	75.11 (27.45)	76.88 (27.37)	68.50 (26.78)	<0.001
Systolic pressure	130.97 (20.85)	132.09 (20.55)	126.81 (21.48)	0.001
Diastolic pressure	75.33 (11.60)	76.34 (11.45)	71.58 (11.39)	<0.001
BUN	17.35 (6.82)	17.13 (6.81)	18.20 (6.82)	0.033
UA	5.44 (1.62)	5.48 (1.62)	5.32 (1.62)	0.182
CR	0.97 (0.64)	0.96 (0.70)	0.98 (0.33)	0.773
CYS	1.10 (0.51)	1.09 (0.53)	1.16 (0.41)	0.048
TG	145.19 (88.66)	156.15 (92.81)	104.35 (54.41)	<0.001
TC	182.94 (36.87)	185.18 (37.63)	174.59 (32.63)	<0.001
HDLC	50.27 (12.17)	49.08 (11.55)	54.69 (13.40)	<0.001
LDLC	101.60 (28.53)	103.21 (28.84)	95.57 (26.54)	<0.001
GLU	105.51 (37.44)	106.69 (38.39)	101.13 (33.39)	0.045
HB	6.11 (1.13)	6.15 (1.13)	5.95 (1.14)	0.017
CRP	3.27 (4.38)	3.31 (4.27)	3.11 (4.78)	0.532

Medians and interquartile ranges (25th and 75th percentiles) were calculated for continuous variables, while frequencies and percentages were determined for categorical variables. The Wilcoxon rank-sum test was used to compare group differences for continuous variables, and Chi-squared tests were employed for categorical variables. BMI mean Body Mass Index; BUN mean Blood Urea Nitrogen; UA mean Uric Acid; CR mean Creatinine; CYS mean Cystatin C; TG mean Triglycerides; TC mean Total Cholesterol; LDLC mean Low Density Lipoprotein-Cholesterol; HDLC mean High Density Lipoprotein-Cholesterol; GLU mean Glucose; HB mean Glycated Hemoglobin; CRP mean C-Reactive Protein.

The cohort exhibited significant gradients in clinical profiles across CKD stages (Supplementary Table S2). Participants with advanced CKD (G3b-G5) were older (mean age 71.6 vs. 60.9 years in G1-G2, *p* < 0.001) and had higher rates of hypertension (58.5% vs. 38.9%, *p* < 0.001) and IADL disability (24.5% vs. 13.0%, *p* = 0.001). Cognitive function declined progressively (8.13 vs. 10.38, *p* < 0.001), paralleled by elevated inflammatory markers (CRP: 4.49 vs. 2.70 mg/L, *p* < 0.001). Sarcopenia prevalence nearly doubled in G3b-G5 (29.9%) compared to G1-G2 (16.2%, *p* < 0.001), underscoring the multifactorial burden in advanced CKD.

The dataset, comprising 1,092 participants, was randomly divided into two subsets: a training set and an internal validation set. To ensure that the two subsets were comparable, we conducted thorough statistical comparisons, the results of which are provided in Supplementary Table S1. These analyses were aimed at evaluating the equivalence of the training and validation cohorts across key demographic and clinical characteristics. The findings revealed no significant differences (*p* > 0.05).

### 3.2 Feature selection using LASSO regression

**Figure 2:**
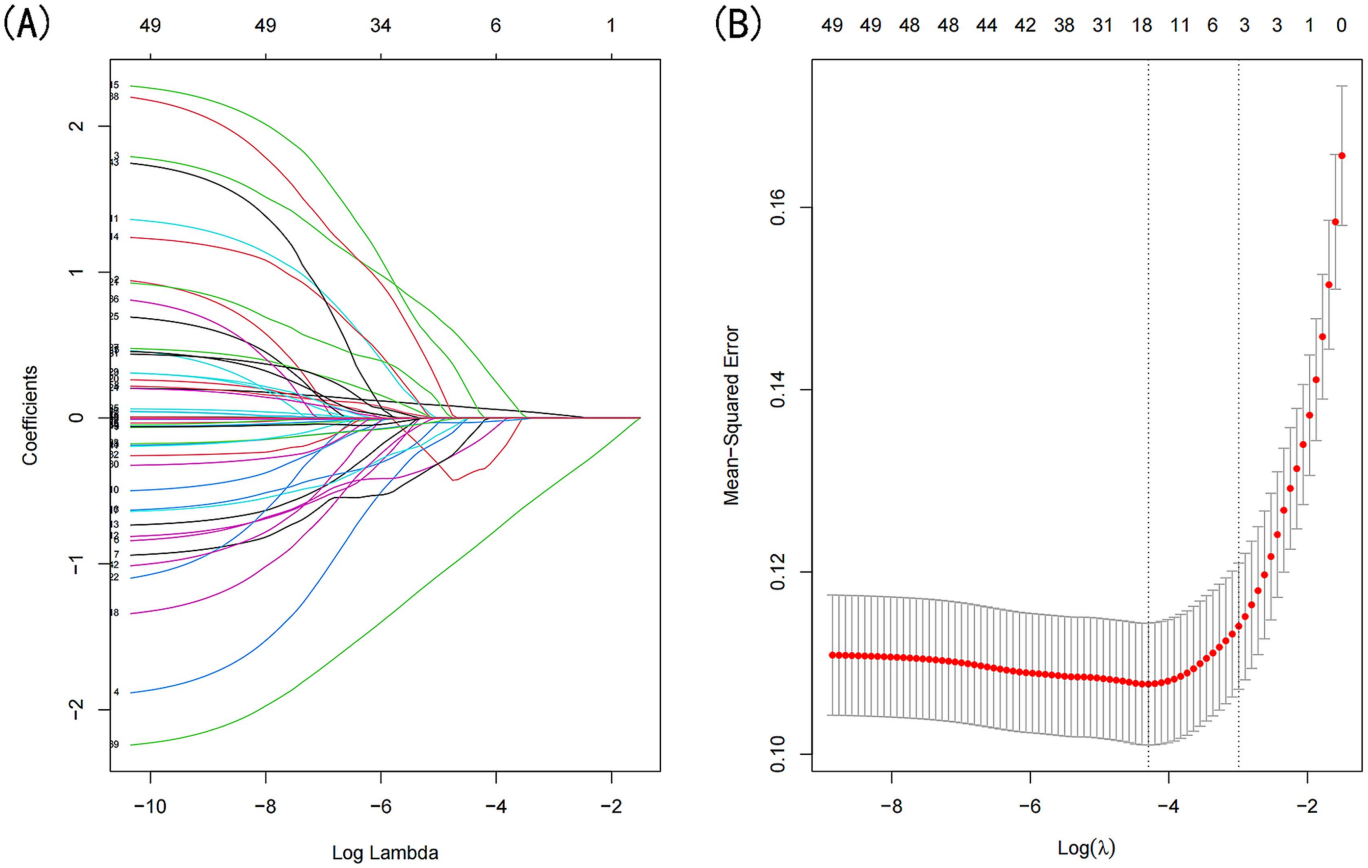
Demographic and clinical feature selection was performed using the LASSO regression model. **(A)** Coefficient profile was generated based on the logarithmic (lambda) sequence, with non-zero coefficients identified at the optimal lambda. **(B)** The optimal lambda parameter was selected through tenfold cross-validation using the minimum criteria. The partial likelihood deviation (binomial deviation) curve relative to log(lambda) was plotted, and a vertical line was drawn at the optimal value using the 1-SE criterion.

LASSO regression was applied to the training dataset (*n* = 764) to identify the most relevant predictors of sarcopenia in CKD patients (Figure 2). The following predictors were retained in the model: age, low-density lipoprotein cholesterol (LDL-C), high-density lipoprotein cholesterol (HDL-C), triglycerides (TG), waist circumference, and diastolic blood pressure (DBP). These variables were subsequently included in the multivariate logistic regression analysis.

### 3.3 Multivariate logistic regression analysis

Multivariate logistic regression was performed to validate the independent predictors of sarcopenia. The results are presented in Table 2. Age: OR = 4.39 (95% CI: 3.08–6.26), LDLC: OR = 0.66 (95% CI: 0.48–0.90), HDLC: OR = 1.40 (95% CI: 1.06–1.84), TG: OR = 0.53 (95% CI-0.37, 0.76), Waist: OR = 0.32 (95% CI: 0.23–0.43), DBP:OR = 0.62 (95% CI: 0.45–0.86) were identified as significant independent predictors of sarcopenia (*p* < 0.05).

**Table 2:** The prediction model with multivariate logistic regression.

Variables	OR (95% CI)	*p*-value
Age	4.39 (3.08, 6.26)	< 0.001
LDLC	0.66 (0.48, 0.90)	0.009
HDLC	1.40 (1.06, 1.84)	0.018
TG	0.53 (0.37, 0.76)	<0.001
Waist	0.32 (0.23, 0.43)	< 0.001
Diastolic pressure	0.62 (0.45, 0.86)	0.005

OR, odds ratio; CI, confidence interval; LDLC mean Low-Density Lipoprotein Cholesterol; HDLC mean High-Density Lipoprotein Cholesterol; TG mean triglycerides.

**Figure 3:**
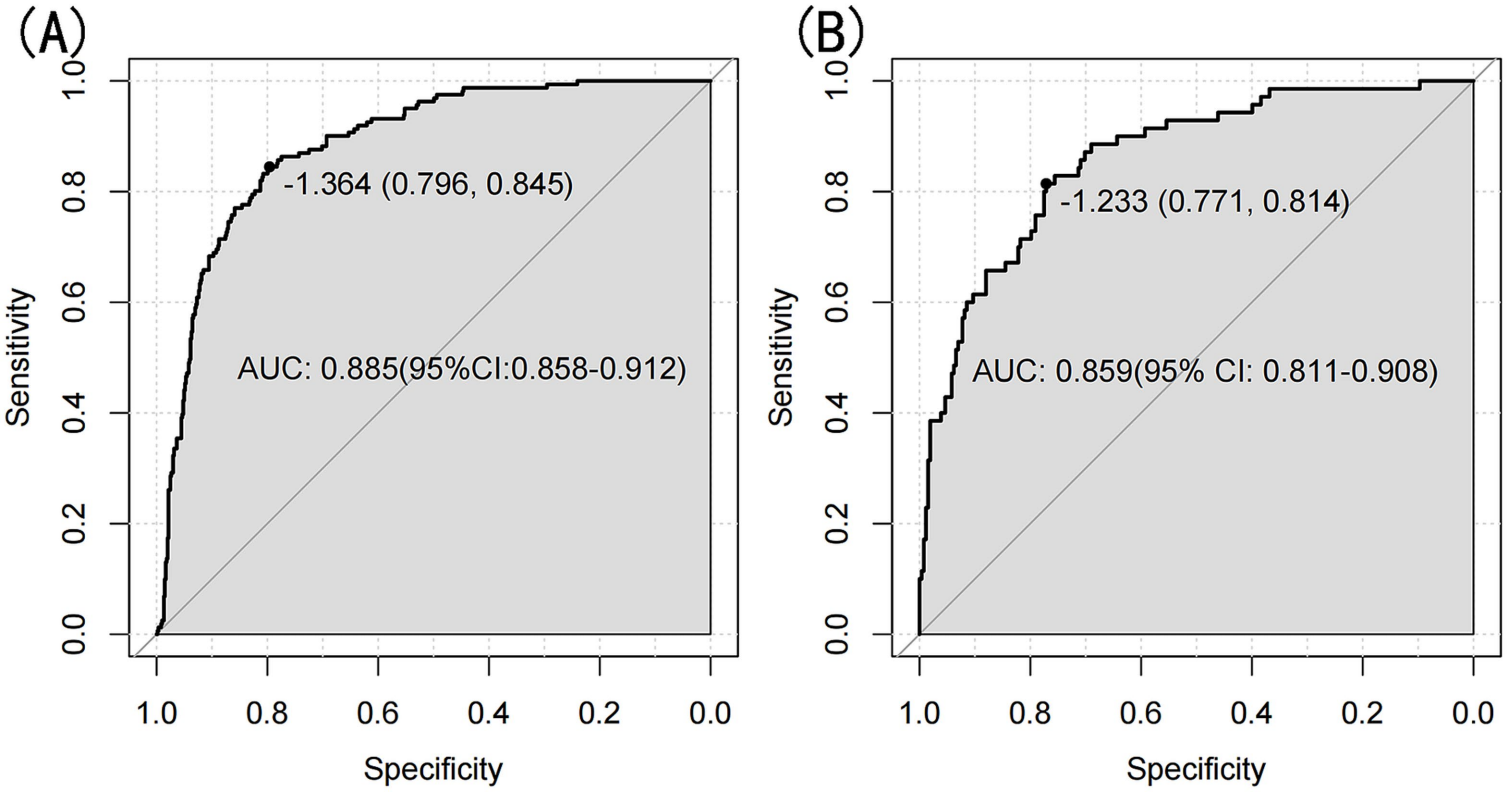
**(A)** Nomogram ROC curves generated from the training set. **(B)** Nomogram ROC curves generated using the validation set.

**Figure 4:**
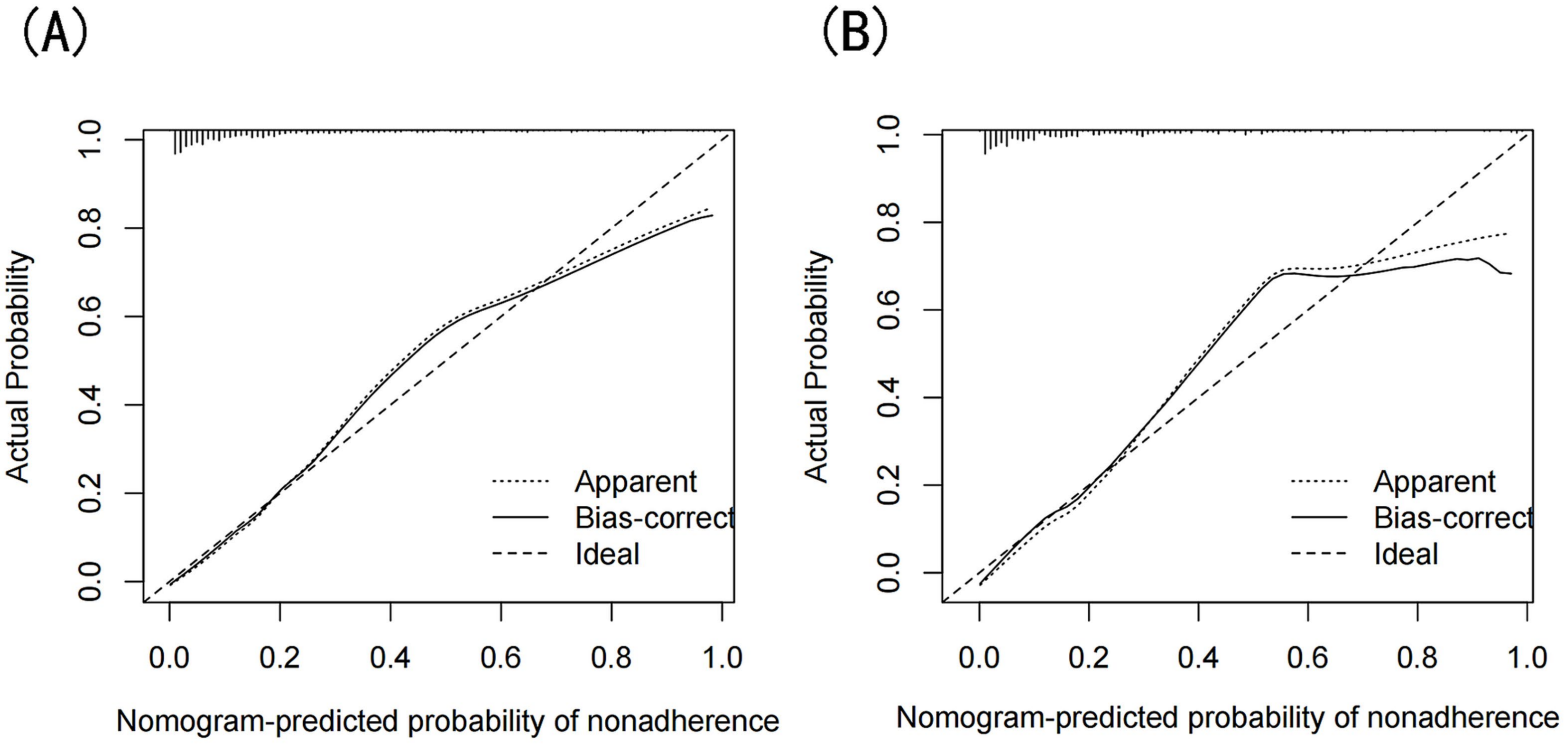
**(A)** Calibration curves of the nomogram prediction for the training set, **(B)** Calibration curves of the nomogram prediction for the validation set.

**Figure 5:**
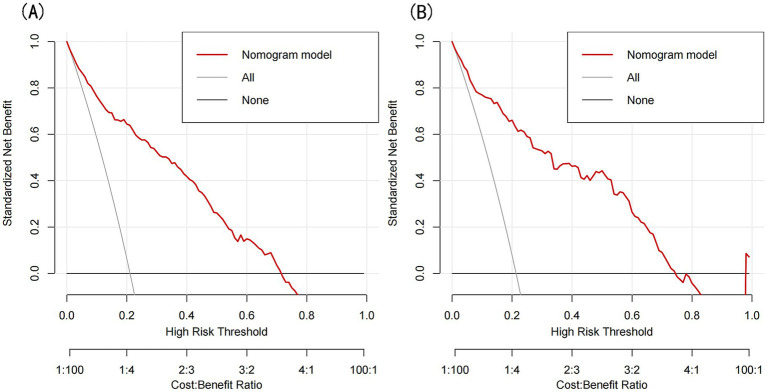
**(A)** DCA curves for the training set, **(B)** DCA curves for the validation set.

The model’s performance was evaluated using several methods, including the area under the receiver operating characteristic curve (AUC) for both the training and validation datasets, as well as calibration curves and decision curve analysis (DCA). In the training set (Figure 3A), the model achieved an AUC of 0.886 (95% CI: 0.858–0.912), while in the validation set (Figure 3B), the AUC was 0.859 (95% CI: 0.811–0.908), demonstrating ability to distinguish between sarcopenia patients and non-patients. The Hosmer-Lemeshow goodness-of-fit test yielded *p*-values greater than 0.05 for both the training set (χ^2^ = 13.302, df = 8, *p* = 0.102) and validation set (χ^2^ = 6.748, df = 8, *p* = 0.564). The calibration curve was near the ideal line, further validating the model’s predictive accuracy (Figure 4). Decision curve analysis was performed to evaluate the clinical utility of the model. DCA was conducted to assess the clinical value of the model, with the resulting curve indicating substantial net benefit across a range of decision thresholds, underscoring its potential utility in clinical practice (Figure 5).

### 3.4 Nomogram development

**Figure 6:**
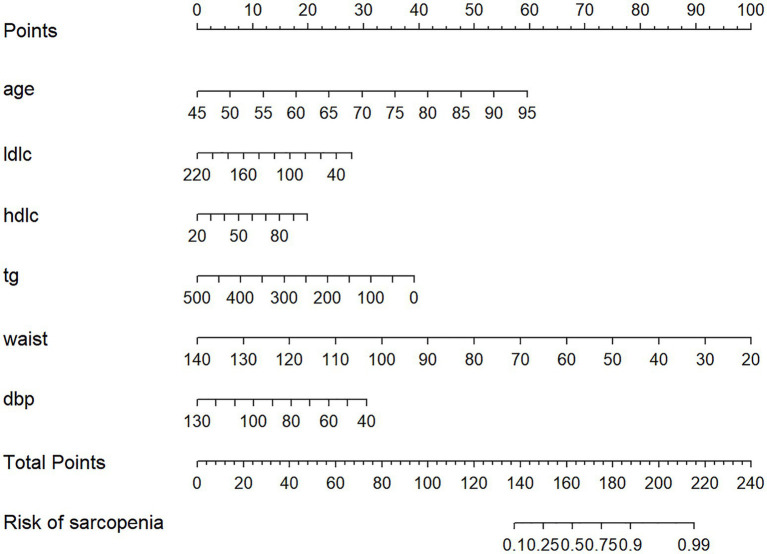
Nomogram to evaluate the risk of sarcopenia in patients with diabetes. LDLC mean low-density lipoprotein cholesterol. HDLC mean high-density lipoprotein cholesterol.TG mean triglycerides.

The nomogram is presented in Figure 6. Each predictor was assigned a score corresponding to its contribution to sarcopenia risk, and the total score was used to estimate the probability of sarcopenia.

### 3.5 Machine learning model performance and comparison

**Figure 7:**
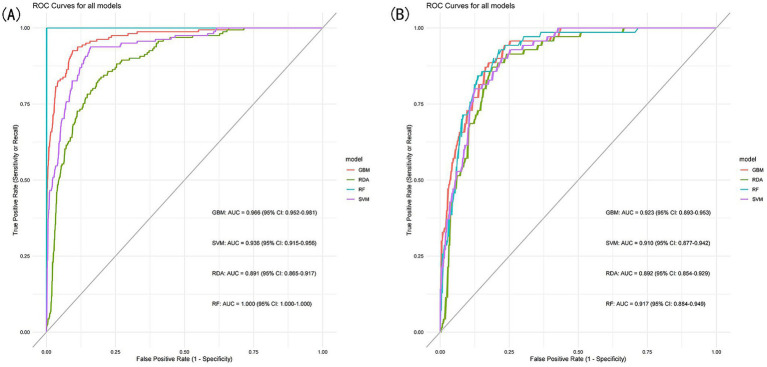
**(A)** ROC curves of the machine learning models for the training set, **(B)** ROC curves of the machine learning models for the validation set.

To further assess the predictive performance and significance of the predictors used in developing the nomogram, we constructed four machine learning models. As shown in Figures 7A,B, these models exhibited strong discriminative ability and predictive value in both the training and validation sets. The Random Forest (RF) model achieved the highest AUC of 1.000 in the training set, but performed moderately in the validation set (AUC = 0.917, 95% CI: 0.884–0.949). In contrast, the Gradient Boosting Machine (GBM) model demonstrated the best predictive performance in the validation set (AUC = 0.923, 95% CI: 0.893–0.953), while ranking second in the training set (AUC = 0.966, 95% CI: 0.952–0.981), just behind the RF model.

### 3.6 Hyperparameter optimization and importance of predictors

**Figure 8:**
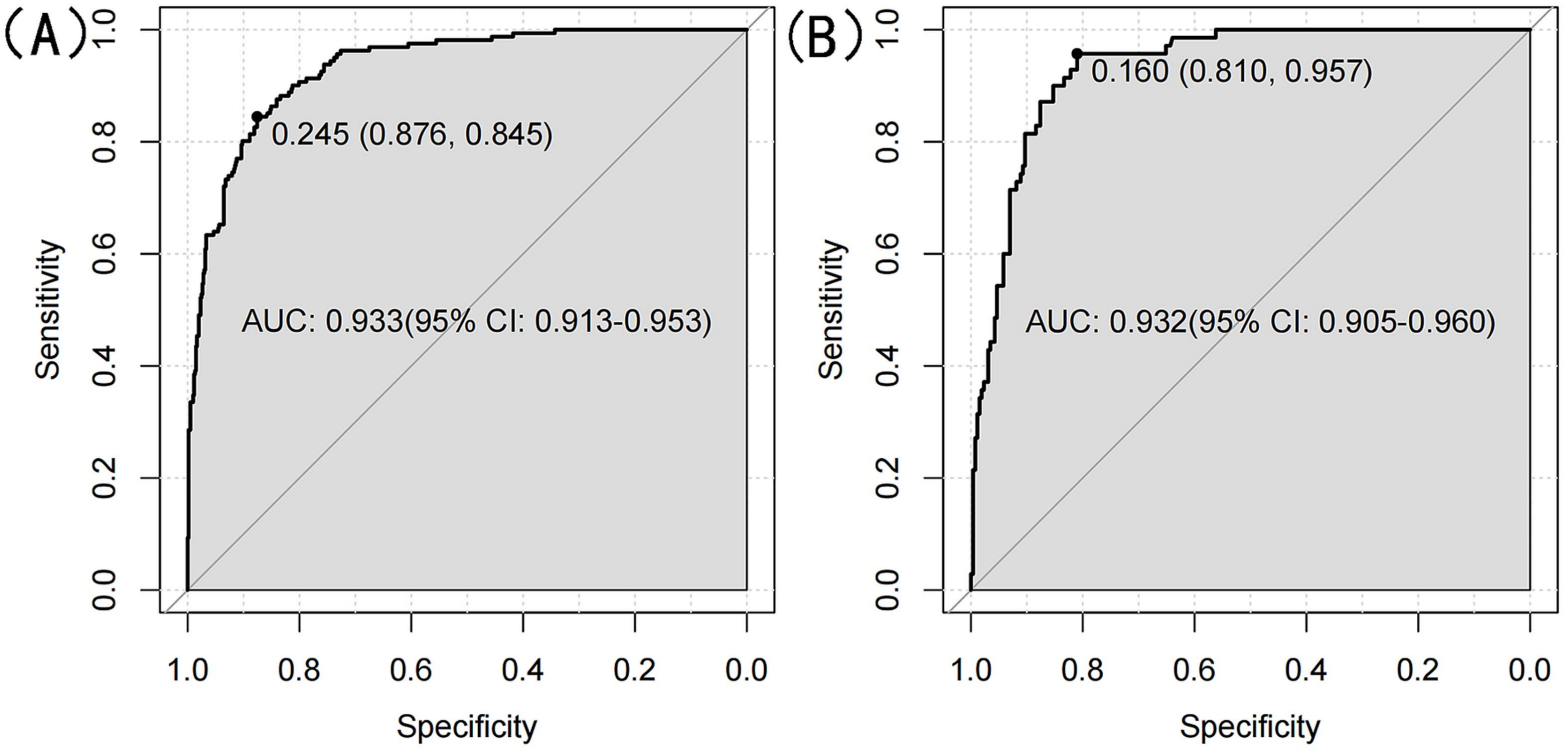
**(A)** ROC curve for optimized GBM model for the training set. **(B)** ROC curve for optimized GBM model for the validation set.

**Figure 9:**
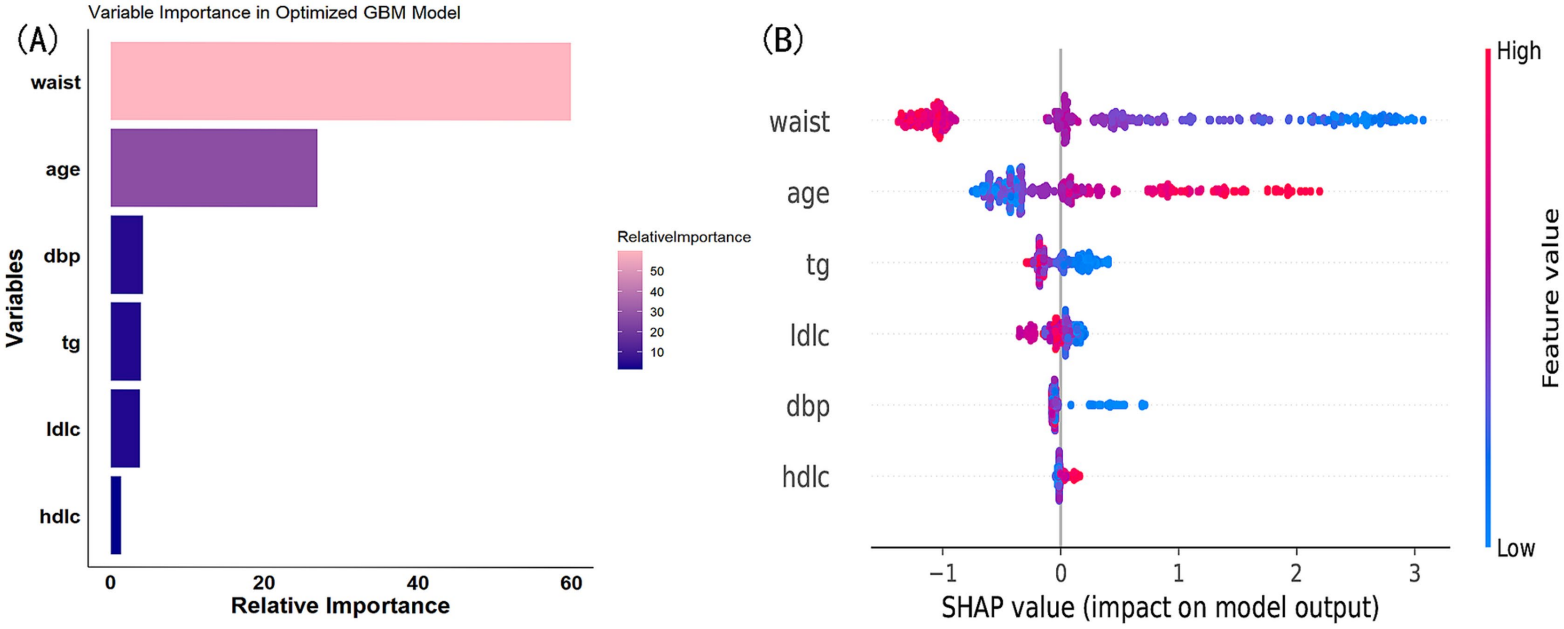
**(A)** Variable importance in optimized GBM model. **(B)** SHAP value contribution in optimized GBM model.

We ultimately selected the Gradient Boosting Machine (GBM) model for hyperparameter optimization using Bayesian optimization. This method is efficient for optimizing hyperparameters by constructing a probabilistic model of the objective function and using it to identify the most promising hyperparameters for evaluation. The optimization process identified the following optimal parameters: 56 trees, an interaction depth of 2, a shrinkage rate of 0.0777, and a minimum number of observations in a node of 30. After optimization, the model achieved an AUC of 0.933 (95% CI: 0.9132–0.9527) for the training set and 0.932 (95% CI: 0.9045–0.9595) for the validation set (Figure 8). Variable importance analysis revealed that waist circumference was the most significant predictor, followed by age, DBP, TG, LDL-C, and HDL-C. Specifically, waist circumference had a relative importance of 59.88%, age 26.92%, DBP 4.22%, TG 3.89%, LDL-C 3.77%, and HDL-C 1.32% (Figure 9A). Additionally, SHAP (Shapley Additive exPlanations) values were used to interpret the model’s predictions. Age and waist circumference were the primary drivers of the model’s output, with other variables contributing to a lesser extent, making smaller adjustments to the final prediction (Figure 9B).

### 3.7 Principal component analysis (PCA) and cluster analysis

**Figure 10:**
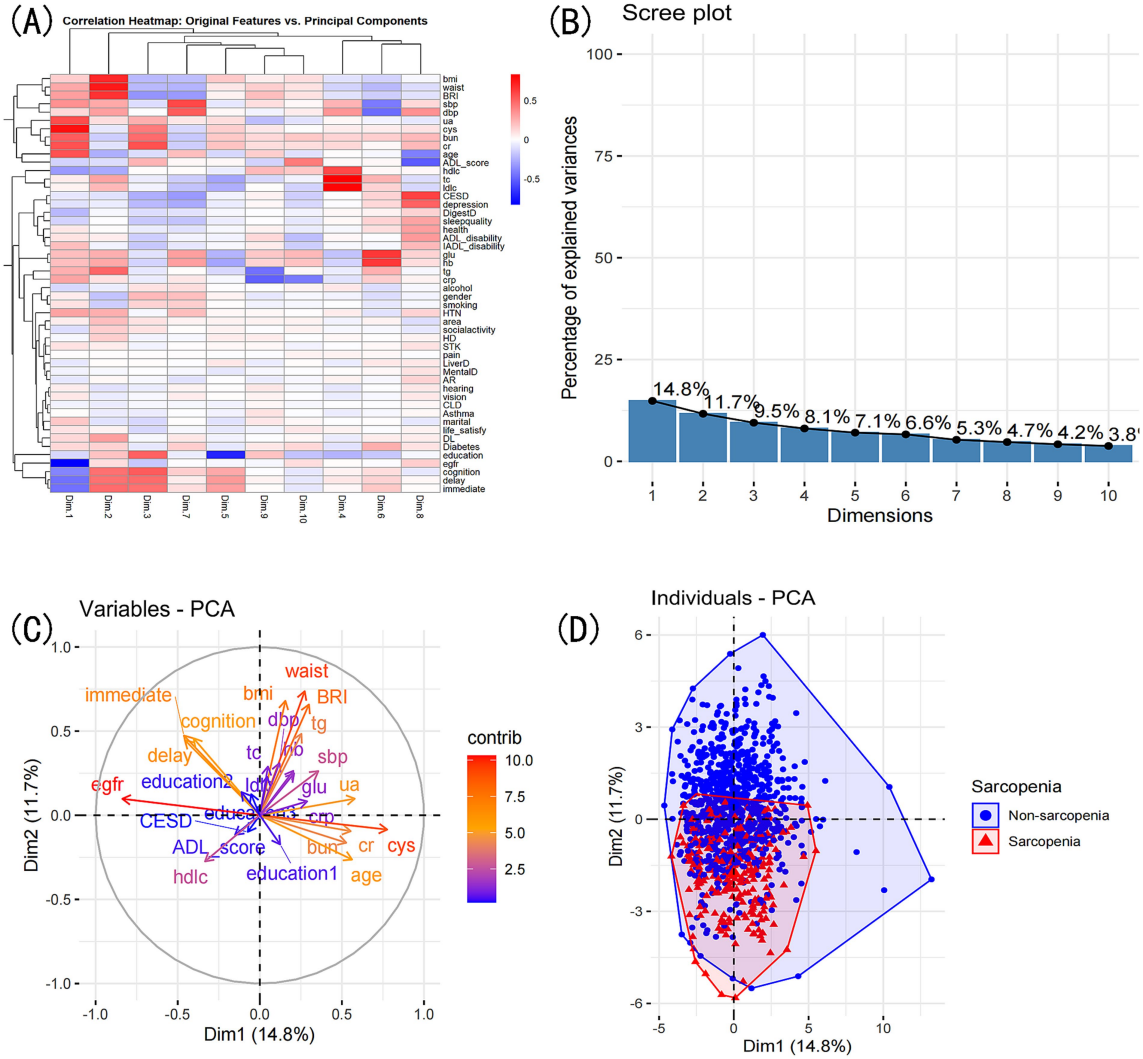
PCA comprehensive analysis. Exploring variable contributions and patient clustering: **(A)** Correlation heatmap of original variables and principal components. **(B)** Top contributing variables for each principal component. **(C)** Grouping of original variables based on positive or negative contributions to PCs. **(D)** Patient grouping based on PCA’s two principal dimensions: scatter plot.

PCA reduced the original 52 features into 10 principal components, explaining a total of 75.8% of the variance. The first two principal components explained 26.5% of the variance. PC1 was most strongly influenced by eGFR, cystatin C, uric acid, age, and creatinine, whereas PC2 was primarily influenced by waist circumference, BMI, BRI, triglycerides, and immediate recall. The PCA biplot (Figure 10) illustrates the contribution of these features. K-means clustering identified four distinct patient subgroups. Cluster 1 had the highest prevalence of sarcopenia (53.4%), while Clusters 2 and 3 had lower prevalence (10.8 and 8.8%, respectively). Among the characteristics available at presentation, the clusters differed significantly across 82.4% (42/51) of all the admission variables recorded (Supplementary Table S3; Supplementary Figure S1).

## 4 Discussion

CKD leads to muscle atrophy through a complex interaction of factors, primarily characterized by a disruption in the balance between protein synthesis and breakdown, along with impaired muscle regeneration (26, 27). A key driver of muscle wasting in CKD is the dysregulation of critical molecular pathways involved in muscle metabolism. The insulin-like growth factor-1 (IGF-1) pathway, which is essential for promoting muscle protein synthesis and cell proliferation, is often disrupted in CKD patients (28). This disruption creates an imbalance between the IGF-1 and myostatin pathways, with myostatin inhibiting muscle growth and promoting protein catabolism (29, 30). Consequently, accelerated catabolic processes contribute to increased muscle atrophy in these individuals (31). Inflammation plays a key role in muscle atrophy in CKD, with increased levels of pro-inflammatory cytokines like tumor necrosis factor-alpha (TNF-*α*) and interleukin-6 (IL-6) driving muscle degradation (32, 33). These cytokines activate the ubiquitin-proteasome system (UPS) and the autophagy-lysosome pathway (ALP), both of which lead to increased proteolysis (34–36). Additionally, oxidative stress from excessive reactive oxygen species (ROS) exacerbates mitochondrial dysfunction, impairing energy production essential for muscle maintenance and repair (37). Other contributing factors include insulin resistance, vitamin D deficiency, and altered levels of angiotensin II, all of which further exacerbate the muscle-wasting process (38, 39). Insulin resistance, common in CKD, diminishes the anabolic effects of insulin on muscle tissue, while vitamin D deficiency is linked to muscle weakness and atrophy (40–44). Moreover, elevated levels of angiotensin II may promote muscle degradation through its effects on inflammation and fibrosis (45, 46).

This study focused on creating and validating a sarcopenia risk prediction model specifically for CKD patients, utilizing data from the 2015 CHARLS cohort. The final model determined that waist circumference, age, LDL-C, TG, HDL-C, and DBP are significant predictors of sarcopenia in CKD patients.

Age emerged as a significant predictor, aligning with the established link between aging and sarcopenia. As individuals age, they experience a progressive decline in muscle mass and strength, driven by several interconnected factors. Hormonal changes, particularly reductions in testosterone, estrogen, and growth hormone, play a crucial role in muscle metabolism and regeneration, leading to decreased muscle protein synthesis and increased muscle breakdown (47, 48). Additionally, the aging process often results in reduced physical activity levels, which further contributes to muscle atrophy and loss of strength. Furthermore, aging is associated with increased oxidative stress and inflammation, both of which can negatively impact muscle health. Elevated levels of reactive oxygen species (ROS) can damage muscle fibers and impair their function (49, 50).

Our findings indicate that in CKD patients, waist circumference, LDL-C, and TC are inversely related to sarcopenia risk, whereas HDL-C is positively associated with an increased risk, whereas high-density lipoprotein cholesterol is positively associated with an increased risk. This finding contrasts with much of the literature, which generally considers abdominal fat a risk factor for muscle loss (51, 52). Many studies have highlighted that excessive body fat, particularly visceral fat, is closely linked to declines in muscle mass, primarily due to the negative effects of inflammatory cytokines secreted by adipose tissue on muscle metabolism (53, 54). However, our results may reflect a protective role of moderate waist circumference and associated fat storage in maintaining muscle health among CKD patients. This hypothesis is further supported by findings from other studies, which showed that patients without sarcopenia had higher waist circumference, LDL-C, and TG levels compared to those with sarcopenia (55). Additionally, in a study constructing a predictive model for sarcopenia, low BMI was identified as a significant risk factor, consistent with findings from a Taiwanese population-based study (16, 56).

Our findings suggest that higher diastolic blood pressure may act as a protective factor against sarcopenia, potentially due to its impact on skeletal muscle perfusion. Chronic low blood pressure can impair blood flow to skeletal muscles, reducing muscle quality and contributing to the progression of sarcopenia. Maintaining adequate blood flow is crucial for delivering oxygen, hormones, and nutrients necessary for muscle maintenance and repair (57). Previous studies have indicated that insufficient perfusion can compromise muscle health, leading to increased muscle loss. Additionally, inadequate capillarization of skeletal muscle restricts the diffusion of substrates, oxygen, hormones, and nutrients, further exacerbating the risk of sarcopenia and the decline in physical function among older adults (58, 59). Consequently, higher diastolic blood pressure levels may reflect better vascular health and more efficient nutrient delivery, ultimately contributing to the preservation of muscle mass and function (60).

A notable strength of this study is the creation of the first nomogram specifically designed to assess sarcopenia risk across the CKD spectrum, leveraging data from the CHARLS. Our model incorporates six commonly available demographic, clinical, functional measures, along with key blood biomarkers such as LDL-C, HDL-C, and triglycerides, enabling simplified risk assessment in community and primary care settings. This broad applicability is particularly valuable given the scarcity of tools for non-dialysis CKD populations. Furthermore, the model’s robust predictive performance was achieved through advanced machine learning methodologies and comprehensive hyperparameter optimization, demonstrating parity with hemodialysis-specific tools despite its broader scope (7, 8).

The clinical applicability of our model is underscored by its capacity to identify CKD patients at elevated risk of sarcopenia through readily obtainable parameters, such as waist circumference, lipid profiles, and blood pressure. Considering the substantial prevalence of sarcopenia among CKD patients and its correlation with adverse clinical outcomes, early detection is imperative for timely and effective intervention. This predictive tool allows clinicians to categorize patients based on their risk and apply targeted strategies, such as personalized nutritional interventions, to improve body composition and support evidence-based dietary practices.

Nevertheless, this study has its limitations. Firstly, the cross-sectional design of the analysis limits our ability to infer causal relationships between the identified predictors and the onset of sarcopenia. Longitudinal research is required to clarify the temporal sequence and mechanistic pathways driving these associations. Second, the relatively modest sample size, while sufficient for model development, may limit the statistical power to detect weaker associations and could potentially influence the stability of the findings. Expanding datasets and conducting collaborative research across multiple cohorts would strengthen the robustness and reliability of the findings. Additionally, the study population was derived exclusively from the CHARLS, which may introduce selection bias and limit the applicability of the findings to other demographic and ethnic groups. External validation in independent CKD cohorts from diverse settings is critical to ensure the model’s generalizability and broader clinical utility. Furthermore, while the model utilizes easily accessible variables, the absence of more sophisticated biomarkers, such as serum myostatin or inflammatory markers, may restrict its ability to capture the complex biological processes driving sarcopenia.

Future studies should aim to increase the sample size and include longitudinal data to enhance causal inference. Integrating advanced omics data and imaging modalities could further refine the predictive accuracy of the model and deepen our understanding of sarcopenia’s pathophysiology. Additionally, while our percentile-based definition of low muscle mass aligns with precedents in aging research and mirrors AWGS DXA thresholds, future studies should harmonize sarcopenia criteria across methodologies to facilitate cross-population comparisons. Finally, clinical implementation research is needed to evaluate the nomogram’s effectiveness in improving patient outcomes, helping to bridge the gap between predictive modeling and real-world healthcare interventions.

## 5 Conclusion

We developed a novel nomogram and machine learning framework for sarcopenia prediction in CKD. The Gradient Boosting Machine model demonstrated optimal performance, providing a validated tool for early risk stratification. This framework offers potential clinical utility in identifying high-risk patients and guiding personalized interventions. Future studies could explore its implementation in clinical practice and further validate its effectiveness in diverse patient populations.

## Data Availability

Publicly available datasets were analyzed in this study. This data can be found at: http://charls.pku.edu.cn.
