# Supplementary material for: Predictive model for sarcopenia in chronic kidney disease: a nomogram and machine learning approach using CHARLS data
Source: Front Med (Lausanne). 2025 Mar 12;12:1546988. doi: 10.3389/fmed.2025.1546988 (PMC11936915; doi:10.3389/fmed.2025.1546988)
Supplement: Supplementary file 1 [file Data_Sheet_1.docx]

Supplementary Material

| **TABLE S1. Comparison between variables in the training and validation datasets.** | | | | |
| --- | --- | --- | --- | --- |
| **Variables** | **Total (n = 1092)** | **Training set (n = 328)** | **Validation set (n = 764)** | **P-value** |
| Age | 65.41 (9.81) | 65.28 (10.06) | 65.47 (9.71) | 0.767 |
| Gender (%) |  |  |  | 0.122 |
| Female | 552 (50.5) | 178 (54.3) | 374 (49.0) |  |
| Male | 540 (49.5) | 150 (45.7) | 390 (51.0) |  |
| Marital (%) |  |  |  | 0.523 |
| Married | 888 (81.3) | 271 (82.6) | 617 (80.8) |  |
| Unmarried | 204 (18.7) | 57 (17.4) | 147 (19.2) |  |
| Education (%) |  |  |  | 0.582 |
| Primary | 1038 (95.1) | 315 (96.0) | 723 (94.6) |  |
| Secondary | 48 ( 4.4) | 11 ( 3.4) | 37 ( 4.8) |  |
| Tertiary education | 6 ( 0.5) | 2 ( 0.6) | 4 ( 0.5) |  |
| Area (%) |  |  |  | 0.153 |
| Rural | 921 (84.3) | 285 (86.9) | 636 (83.2) |  |
| Urban | 171 (15.7) | 43 (13.1) | 128 (16.8) |  |
| Alcohol (%) |  |  |  | 0.386 |
| No | 764 (70.0) | 236 (72.0) | 528 (69.1) |  |
| Yes | 328 (30.0) | 92 (28.0) | 236 (30.9) |  |
| Smoking (%) |  |  |  | 0.022 |
| No | 560 (51.3) | 186 (56.7) | 374 (49.0) |  |
| Yes | 532 (48.7) | 142 (43.3) | 390 (51.0) |  |
| Health (%) |  |  |  | 0.962 |
| Fair | 477 (43.7) | 144 (43.9) | 333 (43.6) |  |
| Good | 87 ( 8.0) | 25 ( 7.6) | 62 ( 8.1) |  |
| Poor | 528 (48.4) | 159 (48.5) | 369 (48.3) |  |
| CESD | 10.93 (7.27) | 10.94 (7.17) | 10.92 (7.32) | 0.971 |
| Depression (%) |  |  |  | 1 |
| No | 541 (49.5) | 162 (49.4) | 379 (49.6) |  |
| Yes | 551 (50.5) | 166 (50.6) | 385 (50.4) |  |
| Hypertension(%) |  |  |  | 0.981 |
| No | 597 (54.7) | 180 (54.9) | 417 (54.6) |  |
| Yes | 495 (45.3) | 148 (45.1) | 347 (45.4) |  |
| Chronic lung diseases(%) |  |  |  | 0.605 |
| No | 843 (77.2) | 257 (78.4) | 586 (76.7) |  |
| Yes | 249 (22.8) | 71 (21.6) | 178 (23.3) |  |
| Cardiovascular disease(%) |  |  |  | 1 |
| No | 739 (67.7) | 222 (67.7) | 517 (67.7) |  |
| Yes | 353 (32.3) | 106 (32.3) | 247 (32.3) |  |
| Stroke(%) |  |  |  | 0.577 |
| No | 1028 (94.1) | 311 (94.8) | 717 (93.8) |  |
| Yes | 64 ( 5.9) | 17 ( 5.2) | 47 ( 6.2) |  |
| Mental disease(%) |  |  |  | 0.512 |
| No | 1067 (97.7) | 319 (97.3) | 748 (97.9) |  |
| Yes | 25 ( 2.3) | 9 ( 2.7) | 16 ( 2.1) |  |
| Arthritis(%) |  |  |  | 0.929 |
| No | 460 (42.1) | 137 (41.8) | 323 (42.3) |  |
| Yes | 632 (57.9) | 191 (58.2) | 441 (57.7) |  |
| Dyslipidemia(%) |  |  |  | 1 |
| No | 832 (76.2) | 250 (76.2) | 582 (76.2) |  |
| Yes | 260 (23.8) | 78 (23.8) | 182 (23.8) |  |
| Liver disease(%) |  |  |  | 0.894 |
| No | 965 (88.4) | 291 (88.7) | 674 (88.2) |  |
| Yes | 127 (11.6) | 37 (11.3) | 90 (11.8) |  |
| Digestive disease(%) |  |  |  | 0.522 |
| No | 660 (60.4) | 193 (58.8) | 467 (61.1) |  |
| Yes | 432 (39.6) | 135 (41.2) | 297 (38.9) |  |
| Diabetes (%) |  |  |  | 0.143 |
| No | 927 (84.9) | 270 (82.3) | 657 (86.0) |  |
| Yes | 165 (15.1) | 58 (17.7) | 107 (14.0) |  |
| Asthma (%) |  |  |  | 0.637 |
| No | 981 (89.8) | 292 (89.0) | 689 (90.2) |  |
| Yes | 111 (10.2) | 36 (11.0) | 75 ( 9.8) |  |
| Socialactivity (%) |  |  |  | 0.473 |
| No | 503 (46.1) | 157 (47.9) | 346 (45.3) |  |
| Yes | 589 (53.9) | 171 (52.1) | 418 (54.7) |  |
| Sleepquality (%) |  |  |  | 0.257 |
| Rarely or none of the time | 424 (38.8) | 120 (36.6) | 304 (39.8) |  |
| Some or a little of the time | 172 (15.8) | 53 (16.2) | 119 (15.6) |  |
| Occasionally or a moderate amount of the time | 181 (16.6) | 48 (14.6) | 133 (17.4) |  |
| Most or all of the time | 315 (28.8) | 107 (32.6) | 208 (27.2) |  |
| ADL_score | 5.85 (0.53) | 5.82 (0.48) | 5.86 (0.54) | 0.273 |
| Cognition | 9.70 (4.22) | 9.51 (4.25) | 9.78 (4.21) | 0.336 |
| Life satisfaction (%) |  |  |  | 0.105 |
| Fair | 662 (60.6) | 213 (64.9) | 449 (58.8) |  |
| Good | 384 (35.2) | 100 (30.5) | 284 (37.2) |  |
| Poor | 46 ( 4.2) | 15 ( 4.6) | 31 ( 4.1) |  |
| Hearing (%) |  |  |  | 0.457 |
| Fair | 624 (57.1) | 194 (59.1) | 430 (56.3) |  |
| Good | 243 (22.3) | 74 (22.6) | 169 (22.1) |  |
| Poor | 225 (20.6) | 60 (18.3) | 165 (21.6) |  |
| Vision (%) |  |  |  | 0.076 |
| Fair | 491 (45.0) | 159 (48.5) | 332 (43.5) |  |
| Good | 318 (29.1) | 80 (24.4) | 238 (31.2) |  |
| Poor | 283 (25.9) | 89 (27.1) | 194 (25.4) |  |
| Pain (%) |  |  |  | 0.786 |
| No | 58 ( 5.3) | 16 ( 4.9) | 42 ( 5.5) |  |
| Yes | 1034 (94.7) | 312 (95.1) | 722 (94.5) |  |
| Waist | 85.98 (14.78) | 85.13 (14.72) | 86.35 (14.80) | 0.212 |
| Height | 157.30 (8.09) | 156.56 (7.89) | 157.61 (8.16) | 0.050 |
| Weight | 59.83 (12.53) | 59.01 (11.40) | 60.17 (12.97) | 0.161 |
| eGFR | 75.11 (27.45) | 76.21 (27.60) | 74.63 (27.38) | 0.384 |
| Systolic pressure | 130.97 (20.85) | 131.92 (21.03) | 130.56 (20.77) | 0.322 |
| Diastolic pressure | 75.33 (11.60) | 76.23 (11.97) | 74.94 (11.42) | 0.094 |
| ADL_disability (%) |  |  |  | 0.11 |
| No | 1043 (95.5) | 308 (93.9) | 735 (96.2) |  |
| Yes | 49 ( 4.5) | 20 ( 6.1) | 29 ( 3.8) |  |
| IADL_disability (%) |  |  |  | 0.648 |
| No | 912 (83.5) | 277 (84.5) | 635 (83.1) |  |
| Yes | 180 (16.5) | 51 (15.5) | 129 (16.9) |  |
| BMI | 24.13 (4.52) | 24.03 (4.06) | 24.18 (4.70) | 0.632 |
| BUN | 17.35 (6.82) | 17.24 (5.70) | 17.40 (7.26) | 0.712 |
| UA | 5.44 (1.62) | 5.41 (1.57) | 5.46 (1.64) | 0.626 |
| CR | 0.97 (0.64) | 0.94 (0.61) | 0.98 (0.65) | 0.378 |
| CYS | 1.10 (0.51) | 1.08 (0.45) | 1.11 (0.53) | 0.317 |
| TG | 145.19 (88.66) | 143.80 (87.48) | 145.79 (89.22) | 0.734 |
| TC | 182.94 (36.87) | 184.04 (40.00) | 182.47 (35.46) | 0.52 |
| HDLC | 50.27 (12.17) | 50.46 (11.86) | 50.19 (12.31) | 0.733 |
| LDLC | 101.60 (28.53) | 102.75 (32.14) | 101.10 (26.84) | 0.383 |
| GLU | 105.51 (37.44) | 103.99 (28.85) | 106.16 (40.58) | 0.38 |
| HB | 6.11 (1.13) | 6.11 (1.28) | 6.11 (1.06) | 0.951 |
| CRP | 3.27 (4.38) | 3.02 (4.09) | 3.37 (4.50) | 0.218 |

**TABLE S2 Baseline Characteristics of the Study Population Stratified by CKD Stage (Cystatin C-Derived eGFR)**

| **Variables** | **Overall** | **G1-G2(≥60)** | **G3a(45-59)** | **G3b-G5(<45)** | **P-value** |
| --- | --- | --- | --- | --- | --- |
| n | 1,092 | 637 | 308 | 147 |  |
| age(mean(SD)) | 65.41(9.81) | 60.91(8.19) | 71.80(7.89) | 71.55(9.16) | <0.001 |
| Gender(male)(%) | 540(49.5) | 309(48.5) | 159(51.6) | 72(49.0) | 0.663 |
| Marital(unmarried)(%) | 204(18.7) | 77(12.1) | 89(28.9) | 38(25.9) | <0.001 |
| Education(%) |  |  |  |  | 0.369 |
| Primary | 1038(95.1) | 600(94.2) | 298(96.8) | 140(95.2) |  |
| Secondary | 48(4.4) | 33(5.2) | 8(2.6) | 7(4.8) |  |
| Tertiary education | 6(0.5) | 4(0.6) | 2(0.6) | 0(0.0) |  |
| Area(urban)(%) | 171(15.7) | 88(13.8) | 58(18.8) | 25(17.0) | 0.123 |
| Alcohol(%) | 328(30.0) | 222(34.9) | 81(26.3) | 25(17.0) | <0.001 |
| Smoking(%) | 532(48.7) | 300(47.1) | 154(50.0) | 78(53.1) | 0.371 |
| Health(%) |  |  |  |  | <0.001 |
| Fair | 477(43.7) | 277(43.5) | 151(49.0) | 49(33.3) |  |
| Good | 87(8.0) | 34(5.3) | 36(11.7) | 17(11.6) |  |
| Poor | 528(48.4) | 326(51.2) | 121(39.3) | 81(55.1) |  |
| CESD(mean(SD)) | 10.93(7.27) | 11.78(7.42) | 9.67(6.81) | 9.85(7.08) | <0.001 |
| Depression(%) | 551(50.5) | 361(56.7) | 133(43.2) | 57(38.8) | <0.001 |
| Hypertension(%) | 495(45.3) | 248(38.9) | 161(52.3) | 86(58.5) | <0.001 |
| Chronic lung diseases(%) | 249(22.8) | 157(24.6) | 62(20.1) | 30(20.4) | 0.228 |
| Cardiovascular disease(%) | 353(32.3) | 223(35.0) | 88(28.6) | 42(28.6) | 0.081 |
| Stroke(%) | 64(5.9) | 30(4.7) | 24(7.8) | 10(6.8) | 0.146 |
| Mental disease(%) | 25(2.3) | 19(3.0) | 4(1.3) | 2(1.4) | 0.193 |
| Arthritis(%) | 632(57.9) | 414(65.0) | 150(48.7) | 68(46.3) | <0.001 |
| Dyslipidemia(%) | 260(23.8) | 153(24.0) | 69(22.4) | 38(25.9) | 0.709 |
| Liver disease(%) | 127(11.6) | 94(14.8) | 20(6.5) | 13(8.8) | 0.001 |
| eGFR(mean(SD)) | 75.11(27.45) | 94.86(16.52) | 53.43(4.22) | 34.92(9.18) | <0.001 |
| Digestive disease(%) | 432(39.6) | 315(49.5) | 79(25.6) | 38(25.9) | <0.001 |
| Diabetes(%) | 165(15.1) | 93(14.6) | 42(13.6) | 30(20.4) | 0.145 |
| Asthma(%) | 111(10.2) | 68(10.7) | 31(10.1) | 12(8.2) | 0.660 |
| Socialactivity(%) | 589(53.9) | 363(57.0) | 160(51.9) | 66(44.9) | 0.021 |
| Sleepquality(%) |  |  |  |  | <0.001 |
| 1 | 424(38.8) | 204(32.0) | 145(47.1) | 75(51.0) |  |
| Rarely or none of the time | 172(15.8) | 104(16.3) | 49(15.9) | 19(12.9) |  |
| Some or a little of the time | 181(16.6) | 109(17.1) | 48(15.6) | 24(16.3) |  |
| Occasionally or a moderate amount of the time | 315(28.8) | 220(34.5) | 66(21.4) | 29(19.7) |  |
| ADL_score(mean(SD)) | 5.85(0.53) | 5.86(0.52) | 5.85(0.48) | 5.78(0.61) | 0.238 |
| Delay(mean(SD)) | 2.45(1.92) | 2.80(1.91) | 2.12(1.80) | 1.61(1.82) | <0.001 |
| Immediate(mean(SD)) | 3.49(1.83) | 3.88(1.69) | 3.08(1.82) | 2.67(1.94) | <0.001 |
| Cognition(mean(SD)) | 9.70(4.22) | 10.38(3.95) | 9.04(4.41) | 8.13(4.37) | <0.001 |
| Life_satisfy(%) |  |  |  |  | 0.139 |
| Fair | 662(60.6) | 392(61.5) | 184(59.7) | 86(58.5) |  |
| Good | 384(35.2) | 213(33.4) | 118(38.3) | 53(36.1) |  |
| Poor | 46(4.2) | 32(5.0) | 6(1.9) | 8(5.4) |  |
| Hearing(%) |  |  |  |  | 0.114 |
| Fair | 624(57.1) | 385(60.4) | 161(52.3) | 78(53.1) |  |
| Good | 243(22.3) | 135(21.2) | 73(23.7) | 35(23.8) |  |
| Poor | 225(20.6) | 117(18.4) | 74(24.0) | 34(23.1) |  |
| Vision(%) |  |  |  |  | 0.072 |
| Fair | 491(45.0) | 302(47.4) | 134(43.5) | 55(37.4) |  |
| Good | 318(29.1) | 188(29.5) | 83(26.9) | 47(32.0) |  |
| Poor | 283(25.9) | 147(23.1) | 91(29.5) | 45(30.6) |  |
| pain(%) | 1034(94.7) | 603(94.7) | 290(94.2) | 141(95.9) | 0.735 |
| Waist(mean(SD)) | 85.98(14.78) | 84.95(14.69) | 87.53(14.22) | 87.21(16.01) | 0.024 |
| SBP(mean(SD)) | 130.97(20.85) | 127.06(18.96) | 136.95(21.94) | 135.38(22.57) | <0.001 |
| DBP(mean(SD)) | 75.33(11.60) | 75.62(11.50) | 74.97(11.24) | 74.81(12.75) | 0.609 |
| ADL_disability(%) | 49(4.5) | 24(3.8) | 14(4.5) | 11(7.5) | 0.146 |
| IADL_disability(%) | 180(16.5) | 83(13.0) | 61(19.8) | 36(24.5) | 0.001 |
| BRI(mean(SD)) | 5.23(1.26) | 5.09(1.21) | 5.42(1.28) | 5.41(1.39) | <0.001 |
| BMI(mean(SD)) | 24.13(4.52) | 24.12(4.19) | 24.17(5.06) | 24.12(4.72) | 0.988 |
| BUN(mean(SD)) | 17.35(6.82) | 15.58(4.32) | 17.83(5.07) | 24.04(12.39) | <0.001 |
| UA(mean(SD)) | 5.44(1.62) | 4.91(1.37) | 5.88(1.56) | 6.82(1.66) | <0.001 |
| CR(mean(SD)) | 0.97(0.64) | 0.80(0.21) | 0.99(0.24) | 1.62(1.48) | <0.001 |
| CYS(mean(SD)) | 1.10(0.51) | 0.83(0.15) | 1.27(0.08) | 1.92(0.89) | <0.001 |
| TG(mean(SD)) | 145.19(88.66) | 138.20(86.57) | 153.62(91.13) | 157.84(90.05) | 0.008 |
| TC(mean(SD)) | 182.94(36.87) | 182.45(34.03) | 184.22(39.28) | 182.41(43.19) | 0.772 |
| HDLC(mean(SD)) | 50.27(12.17) | 52.19(12.00) | 48.41(12.12) | 45.86(11.30) | <0.001 |
| LDLC(mean(SD)) | 101.60(28.53) | 100.57(26.86) | 103.01(29.19) | 103.06(33.72) | 0.374 |
| DLU(mean(SD)) | 105.51(37.44) | 102.67(36.94) | 108.15(36.73) | 112.31(40.00) | 0.006 |
| HB(mean(SD)) | 6.11(1.13) | 6.04(1.22) | 6.20(0.97) | 6.20(1.05) | 0.076 |
| CRP(mean(SD)) | 3.27(4.38) | 2.70(3.78) | 3.86(4.83) | 4.49(5.35) | <0.001 |
| Sarcopenia_(%) | 231(21.2) | 103(16.2) | 84(27.3) | 44(29.9) | <0.001 |

**TABLE S3 Baseline Characteristics of the Study Population Stratified by cluster**

| **Variables** | **Overall** | **Cluster1** | **Cluster2** | **Cluster 3** | **Cluster 4** | **P-value** |
| --- | --- | --- | --- | --- | --- | --- |
| n | 1,092 | 279 | 352 | 407 | 54 |  |
| age(mean(SD)) | 65.41(9.81) | 69.74(9.00) | 70.25(8.36) | 58.77(7.18) | 61.59(9.52) | <0.001 |
| Gender(male)(%) | 540(49.5) | 180(64.5) | 158(44.9) | 169(41.5) | 33(61.1) | <0.001 |
| Marital(unmarried)(%) | 204(18.7) | 69(24.7) | 86(24.4) | 42(10.3) | 7(13.0) | <0.001 |
| Education(%) |  |  |  |  |  | <0.001 |
| Primary | 1038(95.1) | 279(100.0) | 352(100.0) | 407(100.0) | 0(0.0) |  |
| Secondary | 48(4.4) | 0(0.0) | 0(0.0) | 0(0.0) | 48(88.9) |  |
| Tertiary education | 6(0.5) | 0(0.0) | 0(0.0) | 0(0.0) | 6(11.1) |  |
| Area(urban)(%) | 171(15.7) | 24(8.6) | 71(20.2) | 65(16.0) | 11(20.4) | 0.001 |
| Alcohol(%) | 328(30.0) | 99(35.5) | 79(22.4) | 129(31.7) | 21(38.9) | 0.001 |
| Smoking(%) | 532(48.7) | 174(62.4) | 171(48.6) | 157(38.6) | 30(55.6) | <0.001 |
| Health(%) |  |  |  |  |  | 0.055 |
| Fair | 477(43.7) | 117(41.9) | 142(40.3) | 192(47.2) | 26(48.1) |  |
| Good | 87(8.0) | 28(10.0) | 36(10.2) | 22(5.4) | 1(1.9) |  |
| Poor | 528(48.4) | 134(48.0) | 174(49.4) | 193(47.4) | 27(50.0) |  |
| CESD(mean(SD)) | 10.93(7.27) | 11.59(7.48) | 10.07(6.99) | 11.53(7.29) | 8.52(6.92) | 0.001 |
| Depression(%) | 551(50.5) | 153(54.8) | 155(44.0) | 221(54.3) | 22(40.7) | 0.006 |
| Hypertension(%) | 495(45.3) | 83(29.7) | 237(67.3) | 154(37.8) | 21(38.9) | <0.001 |
| Chronic lung diseases(%) | 249(22.8) | 76(27.2) | 68(19.3) | 91(22.4) | 14(25.9) | 0.117 |
| Cardiovascular disease(%) | 353(32.3) | 74(26.5) | 110(31.2) | 154(37.8) | 15(27.8) | 0.014 |
| Stroke(%) | 64(5.9) | 16(5.7) | 29(8.2) | 18(4.4) | 1(1.9) | 0.082 |
| Mental disease(%) | 25(2.3) | 8(2.9) | 4(1.1) | 10(2.5) | 3(5.6) | 0.162 |
| Arthritis(%) | 632(57.9) | 152(54.5) | 197(56.0) | 250(61.4) | 33(61.1) | 0.243 |
| Dyslipidemia(%) | 260(23.8) | 31(11.1) | 116(33.0) | 96(23.6) | 17(31.5) | <0.001 |
| Liver disease(%) | 127(11.6) | 27(9.7) | 28(8.0) | 68(16.7) | 4(7.4) | 0.001 |
| eGFR(mean(SD)) | 75.11(27.45) | 72.23(23.31) | 52.23(17.69) | 96.08(19.29) | 81.00(28.34) | <0.001 |
| Digestive disease(%) | 432(39.6) | 112(40.1) | 100(28.4) | 200(49.1) | 20(37.0) | <0.001 |
| Diabetes(%) | 165(15.1) | 28(10.0) | 78(22.2) | 54(13.3) | 5(9.3) | <0.001 |
| Asthma(%) | 111(10.2) | 32(11.5) | 36(10.2) | 37(9.1) | 6(11.1) | 0.780 |
| Socialactivity(%) | 589(53.9) | 132(47.3) | 168(47.7) | 252(61.9) | 37(68.5) | <0.001 |
| Sleepquality(%) |  |  |  |  |  | <0.001 |
| 1 | 424(38.8) | 121(43.4) | 158(44.9) | 125(30.7) | 20(37.0) |  |
| Rarely or none of the time | 172(15.8) | 27(9.7) | 50(14.2) | 81(19.9) | 14(25.9) |  |
| Some or a little of the time | 181(16.6) | 42(15.1) | 61(17.3) | 70(17.2) | 8(14.8) |  |
| Occasionally or a moderate amount of the time | 315(28.8) | 89(31.9) | 83(23.6) | 131(32.2) | 12(22.2) |  |
| ADL_score(mean(SD)) | 5.85(0.53) | 5.91(0.32) | 5.73(0.74) | 5.90(0.42) | 5.98(0.14) | <0.001 |
| Delay(mean(SD)) | 2.45(1.92) | 1.25(1.44) | 1.89(1.68) | 3.63(1.69) | 3.33(1.82) | <0.001 |
| Immediate(mean(SD)) | 3.49(1.83) | 2.38(1.60) | 3.00(1.68) | 4.55(1.45) | 4.39(1.73) | <0.001 |
| Cognition(mean(SD)) | 9.70(4.22) | 7.30(3.86) | 8.81(4.14) | 11.74(3.33) | 12.45(3.86) | <0.001 |
| Life_satisfy(%) |  |  |  |  |  | 0.243 |
| Fair | 662(60.6) | 170(60.9) | 195(55.4) | 261(64.1) | 36(66.7) |  |
| Good | 384(35.2) | 98(35.1) | 142(40.3) | 129(31.7) | 15(27.8) |  |
| Poor | 46(4.2) | 11(3.9) | 15(4.3) | 17(4.2) | 3(5.6) |  |
| Hearing(%) |  |  |  |  |  | 0.006 |
| Fair | 624(57.1) | 141(50.5) | 195(55.4) | 252(61.9) | 36(66.7) |  |
| Good | 243(22.3) | 61(21.9) | 80(22.7) | 92(22.6) | 10(18.5) |  |
| Poor | 225(20.6) | 77(27.6) | 77(21.9) | 63(15.5) | 8(14.8) |  |
| Vision(%) |  |  |  |  |  | 0.031 |
| Fair | 491(45.0) | 114(40.9) | 153(43.5) | 201(49.4) | 23(42.6) |  |
| Good | 318(29.1) | 79(28.3) | 99(28.1) | 118(29.0) | 22(40.7) |  |
| Poor | 283(25.9) | 86(30.8) | 100(28.4) | 88(21.6) | 9(16.7) |  |
| pain(%) | 1034(94.7) | 263(94.3) | 334(94.9) | 386(94.8) | 51(94.4) | 0.985 |
| Waist(mean(SD)) | 85.98(14.78) | 72.99(17.36) | 93.61(10.70) | 88.09(9.87) | 87.56(10.17) | <0.001 |
| SBP(mean(SD)) | 130.97(20.85) | 123.88(19.91) | 143.13(20.43) | 125.46(17.21) | 129.86(18.93) | <0.001 |
| DBP(mean(SD)) | 75.33(11.60) | 70.33(10.39) | 79.09(11.59) | 75.45(11.06) | 75.72(12.26) | <0.001 |
| ADL_disability(%) | 49(4.5) | 10(3.6) | 24(6.8) | 14(3.4) | 1(1.9) | 0.075 |
| IADL_disability(%) | 180(16.5) | 54(19.4) | 85(24.1) | 38(9.3) | 3(5.6) | <0.001 |
| BRI(mean(SD)) | 5.23(1.26) | 4.21(1.33) | 5.94(1.06) | 5.34(0.90) | 5.01(0.96) | <0.001 |
| BMI(mean(SD)) | 24.13(4.52) | 20.60(2.65) | 26.10(4.66) | 24.99(4.13) | 23.08(3.26) | <0.001 |
| BUN(mean(SD)) | 17.35(6.82) | 17.61(5.86) | 19.79(9.14) | 15.12(3.82) | 16.98(6.00) | <0.001 |
| UA(mean(SD)) | 5.44(1.62) | 5.14(1.45) | 6.53(1.56) | 4.71(1.23) | 5.42(1.65) | <0.001 |
| CR(mean(SD)) | 0.97(0.64) | 0.93(0.25) | 1.18(0.90) | 0.79(0.21) | 1.12(1.33) | <0.001 |
| CYS(mean(SD)) | 1.10(0.51) | 1.09(0.29) | 1.43(0.66) | 0.83(0.19) | 1.09(0.65) | <0.001 |
| TG(mean(SD)) | 145.19(88.66) | 103.58(51.97) | 186.63(105.94) | 136.35(74.94) | 156.74(95.21) | <0.001 |
| TC(mean(SD)) | 182.94(36.87) | 166.31(27.86) | 194.25(41.36) | 184.82(34.26) | 181.08(35.43) | <0.001 |
| HDLC(mean(SD)) | 50.27(12.17) | 53.03(12.54) | 45.99(10.92) | 52.55(12.05) | 46.73(10.43) | <0.001 |
| LDLC(mean(SD)) | 101.60(28.53) | 89.78(22.12) | 108.91(31.31) | 103.37(27.61) | 101.60(27.20) | <0.001 |
| DLU(mean(SD)) | 105.51(37.44) | 100.91(38.72) | 113.09(36.33) | 101.85(35.99) | 107.51(41.65) | <0.001 |
| HB(mean(SD)) | 6.11(1.13) | 5.90(1.03) | 6.38(1.18) | 6.01(1.13) | 6.18(1.00) | <0.001 |
| CRP(mean(SD)) | 3.27(4.38) | 2.94(4.49) | 5.03(5.47) | 2.06(2.37) | 2.52(3.92) | <0.001 |
| Sarcopenia_(%) | 231(21.2) | 149(53.4) | 38(10.8) | 36(8.8) | 8(14.8) | <0.001 |

**
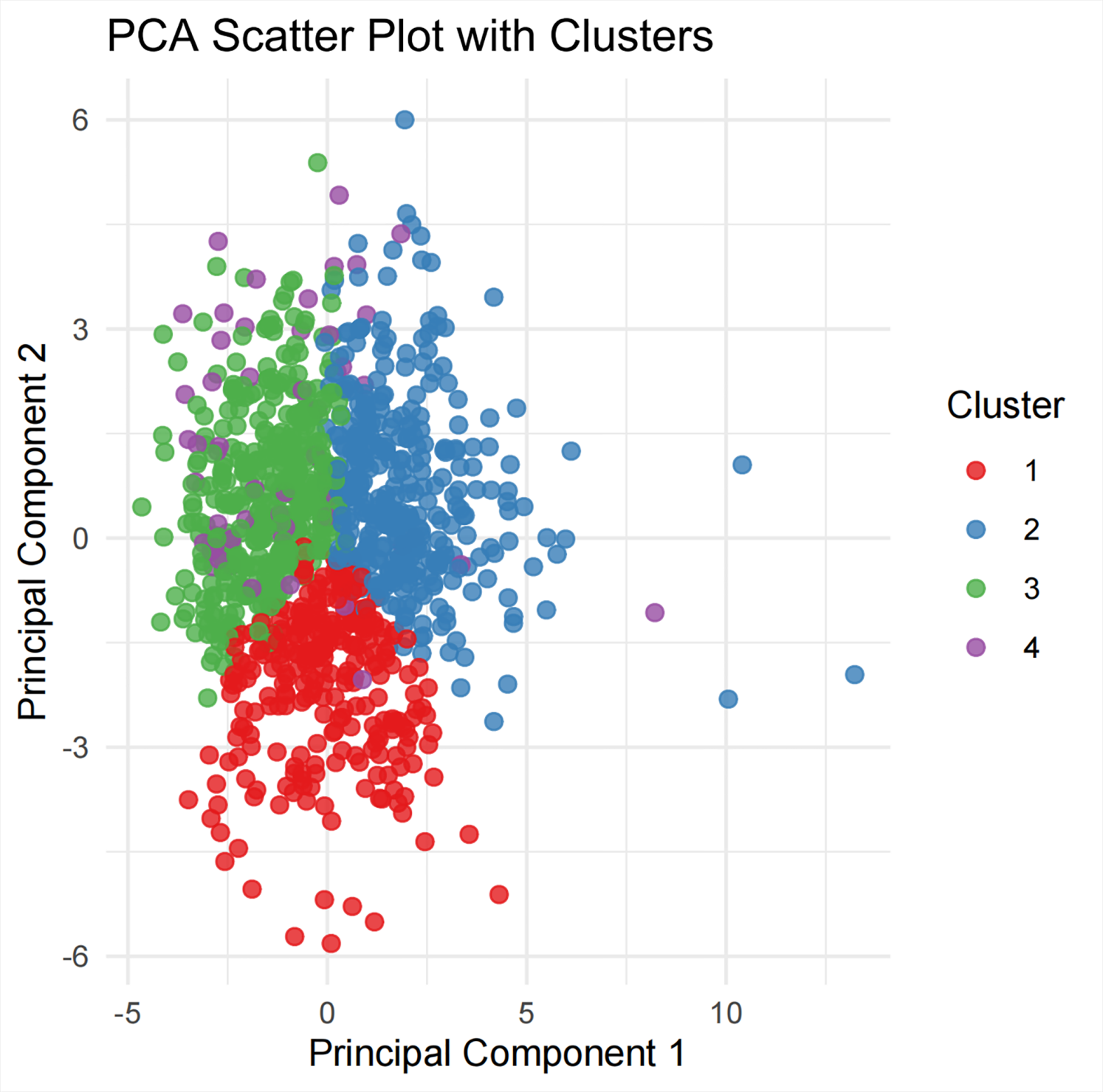
**

**Figure S1** K-means Clustering of Patients Based on Principal Component Analysis
